# Oncology Workforce Crisis: Let’s Work Smarter, Not Harder

**DOI:** 10.3390/curroncol30120758

**Published:** 2023-12-11

**Authors:** Genevieve Chaput

**Affiliations:** Division of Supportive and Palliative Care, McGill University Health Centre, Montreal, QC H4A 3J1, Canada; genevieve.chaput@mcgill.ca

Editorial: Cancer projections in Canada are bleak, with the average annual number of new cancer diagnoses expected to be 79% higher in 2028–2032 compared to 2003–2007 [1]. In this year alone, the Canadian Cancer Society estimates that 27 Canadians will be diagnosed with cancer every hour [2]. Undoubtedly, these escalating projections will translate into an increased need for cancer-related services across Canada [2]. In addition to population growth and aging, without enhancement of diagnostic and treatment capacity, COVID-19-related disruptions to cancer care may adversely impact Canadian cancer deaths over the next few years [3].

The anticipated oncology workforce shortage further complexifies the growing need for cancer care provision nationwide. Medical oncologists’ workload, infrastructure, and care delivery have exceeded the median target of 175 new consults/metric year in over 50% of these specialists [4]. Supply and demand model projections indicate that there is a need for significant growth in the medical oncology workforce to address the steep increase in demand for cancer care [5]. The evolving landscape of oncology therapeutics and longer duration of treatments have contributed to this strain [6]. Radiation oncology workforce projections for 2020–2040 also forecast a deficit in these providers by 2026 [7]. Finally, the Canadian family physician shortage, indicated by predictions that up to 20% of family physicians intend to retire in the next 5 years and by the consistent reduction in medical students selecting family medicine as their residency training in recent years, further raise the flag concerning the anticipated oncology workforce deficits [8].

In this context, urgent and innovative strategic planning by all stakeholders, including frontline oncology providers and patients, is warranted to ensure optimal cancer care provision over the next decades. Care trajectories and resource allocations merit revaluation, with a focus on further development of interspecialty approaches to deliver high-quality oncology care while also limiting the burden on healthcare providers. This might include greater shift of medical oncologists to focus on complex cases that require specialized resources and of general practitioners in oncology (GPOs) to take on cancer patients that can be treated within community settings. GPO-led oncology services, such as those pioneered in Manitoba and British Columbia, have proven effective in delivering cancer treatments to patients within the proximity of their homes [9,10].

GPOs, also known as family practitioners in oncology, are family physicians with focused practices in oncology [11]. Through their collaboration with oncology specialists, GPOs across Canada provide meaningful contributions to the care of cancer patients in both in-patient and out-patient settings [12]. In 2022, through the Canadian Association of General Practitioners in Oncology (CAGPO), Dr. Mary DeCarolis and I led a nationwide survey targeting GPOs. Of 100 responders, cumulative responses indicated that 61.5% provide chemotherapy and/or systemic therapy, 11.5% work in close collaboration with radiation oncologists, and 33% and 78% actively provide survivorship and supportive/palliative care, respectively. Our survey also highlighted GPOs’ contributions to the care of a wide range of cancer populations, including breast, lung, gastrointestinal, genitourinary, hematological, head and neck, skin/melanoma, central nervous system, and gynecological cancer patients. These findings reinforce GPOs’ invaluable contribution and their undeniable potential to provide oncology care to Canadians for years to come [12,13].

To support oncology providers through this changing landscape and the growing need for cancer care provision, providing high-quality, evidence-based educational opportunities is essential. To this end, CAGPO is pleased to partner with Current Oncology for its fourth educational six-article series. Through the invaluable contribution of selected experts, this series will cover up-to-date and practical evidence on relevant oncology and supportive care topics. Having recently reached its 20-year milestone, CAGPO’s commitment to supporting the educational needs of GPOs and other oncology professionals is unwavering. To learn more about CAGPO and its membership benefits, please visit https://www.cagpo.ca/membership.
